# Young Black Women’s Breast Cancer Knowledge and Beliefs: A Sequential Explanatory Mixed Methods Study

**DOI:** 10.1007/s40615-024-02208-5

**Published:** 2024-10-22

**Authors:** Chinenye Ilodianya, Michelle S. Williams

**Affiliations:** 1 Academy of Science, 42075 Loudoun Academy Drive, Leesburg, VA 20175 USA; 2 Department of Global and Community Health, College of Public Health, George Mason University, Peterson Hall Room #4505, 4400 University Boulevard, Fairfax, VA 22030 USA

**Keywords:** Breast cancer, Mixed methods, Risk perceptions, Cancer beliefs, Black women

## Abstract

**Introduction:**

Black women under the age of 50 have a 111% higher breast cancer mortality rate than their White counterparts. The breast cancer mortality disparities among young Black women may be due in part to the fact that they are more likely to be diagnosed with late-stage, invasive breast cancer tumors. Psychosocial factors, such as lack of perceived risk for breast cancer, lack of awareness of breast cancer risk factors, and ambiguity about breast cancer screening guidelines are areas that are under investigated among young Black women. The purpose of this study was to identify young Black women’s cancer beliefs and level of breast cancer risk knowledge.

**Methods:**

A sequential explanatory mixed methods study was conducted using quantitative data from the Health Information Trends Survey 6 (HINTS 6) (*n* = 25) and qualitative data from interviews with young Black female college students (*n* = 13). The results of the quantitative data analysis were used to guide the development of the qualitative interview guide. Data regarding participants’ cancer beliefs, cancer risk factor knowledge, perceived cancer risk, and ambiguity about cancer screening behaviors were analyzed.

**Results:**

The findings indicated young Black women have low perceived risk of developing cancer. Most participants were not aware of cancer recommendations that were targeted towards women under the age of 40. In addition, knowledge about lifestyle behavior risk factors for breast cancer was relatively low.

**Conclusion:**

Our findings underscore the importance of developing, disseminating, and implementing breast cancer education interventions that are targeted towards young Black women.

## Introduction

In the US, breast cancer is the most commonly diagnosed cancer among Black women and White women [1]. Overall, the incidence rate of breast cancer among Black women has remained relatively stable over the last decade, while the incidence of breast cancer among White women has increased [2–4]. Although White women have a higher lifetime probability of developing breast cancer, Black women have a higher lifetime probability of dying from breast [1, 3, 4]. The breast cancer mortality rate is 40% higher among Black women aged 50 and older and 111% higher among Black women under the age of 50 [1, 3–5]. Disparities in breast cancer mortality rates among Black women may be due in part to the fact that they are more likely to be diagnosed with late-stage, invasive breast cancer tumors [1, 3, 6]. Women who are diagnosed with early-stage, localized breast cancer tumors have significantly better overall survival rates than women who are diagnosed with regional or distant tumors [1, 7]. For example, the 5-year survival rate of Black women under the age of 50 who are diagnosed with breast cancer at stage I is nearly 4 times higher than that of Black women who are diagnosed with breast cancer at stage IV [8]. In addition, young Black women have a higher risk of being diagnosed with triple-negative breast cancer, which is more aggressive and has high metastatic potential [9, 10]. Therefore, more attention needs to be focused on reducing late-stage breast cancer diagnosis among young Black women.

Psychosocial factors that may contribute to young Black women presenting with later-stage breast cancers include the lack of knowledge about breast cancer, lack of perceived risk for breast cancer, the lack of knowledge about breast cancer risk factors, and the lack of awareness about age-appropriate and risk-appropriate breast cancer screening guidelines [11–16]. Previous studies have found that a higher perceived risk of developing breast cancer, an internal locus of control, and fatalistic cancer beliefs are associated with adherence to breast cancer screening guidelines [17–19]. However, there is a gap in the research about the association between these psychosocial factors and breast cancer outcomes among Black women under the age of 40.

Evidence has shown that cancer education interventions are most effective at increasing cancer screening intentions and uptake when they are culturally relevant and targeted towards a specific audience [20, 21]. Despite persistent disparities in breast cancer outcomes among Black women under the age of 40, very few breast cancer education interventions are targeted towards them. Understanding young Black women’s cancer risk perceptions and knowledge of breast cancer risk factors is necessary to develop targeted breast cancer education interventions.

The purpose of this study was to identify young Black women’s cancer beliefs and level of cancer risk factor knowledge. We conducted a sequential explanatory mixed-methods study using quantitative data from the Health Information Trends Survey 6 (HINTS 6) and qualitative data from interviews with young Black female college students. Quantitative and qualitative data analysis were conducted to identify key breast cancer risk perceptions, breast cancer knowledge deficits, and preferred cancer communication strategies among the target population.

## Methods

The study was conducted between June and August of 2023. A sequential explanatory mixed methods study design was used. Quantitative data from the HINTS 6 was analyzed during the first phase of the study. An interview guide was developed based on the findings of the quantitative data analysis and constructs of the Theory of Planned Behavior. During the second phase of the study, the interview guide was used to conduct in-depth interviews with young Black women to confirm the quantitative findings.

The HINTS 6 was a cross-sectional, nationally representative survey that was administered by the National Institutes of Health (NIH) National Cancer Institute (NCI) between March and November 2022 [22]. The NCI uses the HINTS to assess cancer communication trends among non-institutionalized, civilian adults aged 18 and older [22, 23]. The HINTS 6 was selected as the quantitative data source for this study because it included questions focused on cancer beliefs and the sampling strategy included an increase in addresses in rural areas with high concentrations of minority adults [22]. The HINTS 6 protocol was reviewed and approved by the Westat Institutional Review Board and the NIH Office of Human Subjects Research Protections [23].

The sample for the quantitative phase of our study only included individuals who identified as non-Hispanic Black women (*n* = 25) or non-Hispanic White women (*n* = 104) between the ages of 18 and 26. Data from the sociodemographic section included, age, race/ethnicity, health insurance status, rural/urban designation, sexual orientation, household income, marital status, highest level of education, and occupation. Data for 15 questions in the *Beliefs about Cancer* section of the HINTS 6 were used. The *Beliefs about Cancer* questions were designed to assess cancer risk perceptions including perceived risk of developing cancer, knowledge of cancer risk factors, and confusion about cancer prevention recommendations [23]. For example, the question “How worried are you about getting cancer?” was used to assess the perceived risk of developing cancer. The question “How much do you agree/disagree that there’s not much you can do to lower your chances of getting cancer?” was used to measure cancer fatalism. Knowledge of cancer risk factors was assessed by the following questions: “How much do you think that eating too much fast food could increase a person’s chance of developing cancer?” and “How much do you think that drinking alcohol could increase a person’s chance of developing cancer?” Confusion about cancer prevention recommendations was assessed by the question “There are so many different recommendations about preventing cancer, it’s hard to know which ones to follow….”

Fisher’s exact probability tests were conducted to determine if there were any associations between the participants’ race and their cancer risk perceptions. Statistical significance was determined by an alpha of 0.05. IBM SPSS Statistics version 17 was used to conduct the quantitative data analysis.

For the qualitative phase of the study, in-person interviews were conducted over a 1-week period in July 2023 on the campus of a large public university in the Southeast region of the US. Women who identified as Black or African American and were between 18 and 26 were eligible to participate in the study. Qualitative phase participants (*n* = 13) completed a screening assessment to determine their eligibility. Eligible participants provided verbal informed consent before completing a brief demographic survey. Next, the interviewer used the semi-structured interview guide to conduct an interview in a private area in a building on the college campus. Questions in the interview guide were based on findings from the analysis of the HINTS 6 data. After the quantitative data were analyzed, the research team members determined which cancer risk perceptions required additional data. For example, “What do you think can be done to prevent or reduce the risk of developing breast cancer?” was used to further assess health beliefs, and “What are the recommendations for breast cancer screening tests?” was used to further assess confusion among cancer prevention recommendations. All interviews were conducted in-person by a Black woman who was close in age to the participants. The interviews lasted between 10 and 15 min. After the interview, participants were given a gift card as an honorarium. Data collection was stopped once data saturation was reached. The protocol for the qualitative phase was approved by the Institutional Review Board at a large public university in the Southeast region of the US.

The qualitative data was stored and organized in ATLAS.ti. Two members of the research team coded each transcript first using open coding. Thematic coding was performed for the second cycle of coding. The research team members met to review the coding and to establish interrater reliability. The codes were organized into themes according to cancer risk perceptions that emerged during the analysis.

For the final analysis, the quantitative data and qualitative data were merged. This included observing similarities between responses from the HINTS 6 survey and responses from the in-person interviews in order to better understand breast cancer risk perceptions among young Black women.

## Results

### Participant Characteristics

The demographic characteristics of the HINTS 6 participants are displayed in Table 1. The sample from the HINTS 6 consisted of young Black women with a mean age of 23.08 ± 2.382 years and young White women with a mean age of 22.44 ± 2.557 years. None of the young Black women responders had a personal history of cancer, whereas 3.8% of the young White women responders had a personal history of cancer. Over 50% of all the participants had attended college. The young Black women in the sample were significantly less likely to have health insurance than the young White women (23% vs 6%, respectively, *p* < 0.05). The qualitative phase participants had similar demographic characteristics. The mean age of the qualitative phase was 19.88 ± 1.42 years. All of the qualitative phase participants were enrolled in college at the time of the interview.

**Table 1:** Sociodemographic characteristics of the HINTS 6 participants

Characteristic	Participants’ race		*p*-value
	Black *n* (%)	White *n* (%)	
Age, mean ± SD	23.08 ± 2.382	22.44 ± 2.557	0.479
Rural/urban designation			0.155
Metro county	25 (100)	95 (90.5)	
Nonmetro county	0 (0)	9 (8.57)	
Health insurance			0.044*
No	5(22.7)	5 (6.7)	
Yes	17 (77.3)	70 (93.0)	
Sexual orientation			0.121
Heterosexual, or straight	16 (64)	73 (68.9)	
Homosexual, or gay or lesbian	0 (0)	5 (4.7)	
Bisexual	9 (36)	20 (18.9%)	
Something else	0 (0)	8 (7.5)	
Household income			0.091
Less than $20,000	5 (0.20)	24 (22.9)	
$20,000 to < $35,000	7 (0.28)	8 (7.5)	
$35,000 to < $50,000	4 (0,16)	20 (18.9)	
$50,000 to < $75,000	6 (0,24)	25 (18.9)	
$75,000 or More	3 (0.12)	29 (27.4)	
Marital status			0.116
Living as married	2 (8)	23 (21.7)	
Divorced	1 (4)	1 (0.9)	
Widowed	0 (0)	2 (1.9)	
Single, never married	21(84)	63 (59.4)	
Married	1 (4)	17 (16)	
Highest level of education			1.000
Less than high school	0 (0)	5 (4.7)	
High school graduate	4 (16)	23 (21.7)	
Post high school training	2 (8)	4 (3.8)	
Some college	8 (32)	30 (28.3)	
College graduate or more	11 (44)	44 (41.5)	
Personal cancer history			0.395
Yes	0 (0)	3 (2.8)	
No	25 (100)	103 (97.2)	

^*^p-value < .05

### Perceived Risk of Developing Cancer

The results of our study indicated that the Black HINTS 6 participants had a lower perceived risk of developing cancer in their lifetime than the White participants (*p*-value 0.033). The qualitative findings (Table 2) support the belief that cancer risk is based mainly on genetics. For example, a majority of the qualitative phase participants suggested that genetics was the major cancer risk factor. For example, some participants believed that genetics can determine whether someone is more likely to develop breast cancer, emphasizing the importance of “checking genetic markers” to see if breast cancer has occurred in the family.

**Table 2:** Major themes and sample quotes from the qualitative data

Theme	Sample quotes from participants
Being healthy prevents breast cancer	When participants were asked how to prevent/reduce their risk of breast cancer: • “Eating healthier and taking care of yourself …” • “…making sure health and wellness is okay both mentally and physically reduces risk of breast cancer.” • “…living a healthy lifestyle…” When a participant was asked about their chances of developing breast cancer: • “I don’t exercise, and I eat junk food, so probably high.”
Genetics plays a role in breast cancer risk	When participants were asked how to prevent/reduce their risk of breast cancer: • “…Checking genetic markers if family has had breast cancer before…” • “See a genetic counselor.” When participants were asked what causes breast cancer: • “Genetics…”
The importance of regular screening	• “Make sure you get screenings often enough…” • “Get consistently checked so the cancer can be stopped early on.” • “…Increase access to testing cancers for breast cancer so people can catch it early on.”
Environment contributes to risk of breast cancer	When participants were asked what causes breast cancer: • “Being around more radiation…”
Spreading awareness is key	• “Spread knowledge about who is at risk for breast cancer and what causes it…”
Lack of knowledge	When some participants were asked what they know breast cancer screening tests and the recommendations for them: • “Nothing.” • “Not sure.” • “No idea.”
Misinformation	When asked about the recommendations for breast cancer screening tests: • “Around every 10 years.” When asked about what may reduce one’s risk of developing breast cancer: • “Not wearing your bra to sleep…” • “…using more natural products when it comes to hair and skincare.”

### Confusion and Misinformation About Cancer Prevention Recommendations

The quantitative data analysis revealed that many of the Black respondents (68%) and White respondents (64%) believed that it was difficult to know which recommendations to follow when trying to prevent cancer (*p*-value:0.774). Similarly, a lack of knowledge among interview participants about breast cancer risk factors and screening was very common. The findings from the qualitative phase participants confirm that young women’s knowledge of breast cancer screening recommendations is low. Many qualitative phase participants discussed the importance of being regularly screened for breast cancer. Several participants believed it was important that people were “consistently checked so the cancer can be stopped early on.” However, the majority of the qualitative phase participants were unsure what the breast cancer screening recommendations were, even if they were able to provide the name “mammogram” as the test they should be getting at a certain age to protect themselves. In addition, only a few qualitative phase participants mentioned “self-checking” as a way of protecting themselves from dying from breast cancer. Some participants provided incorrect information when asked about breast cancer screening recommendations for screening. For example, one participant stated that women should get a breast cancer screening “every 10 years.”

### Cancer Fatalism

There were no statistically significant differences in cancer fatalism between the young Black HINTS 6 participants and their White counterparts (*p*-value 0.11). About 68% of the young Black women who participated in the HINTS 6 believed there was not much that could be done to decrease their chances of developing cancer. In addition, 59.1% of the Black HINTS 6 participants agreed or strongly agreed “When I think about cancer, I automatically think about death.” This finding was confirmed by the qualitative data. Several of the qualitative phase participants mentioned that factors beyond one’s control, like genetics and the environment one lives in, can be possible contributions to one’s higher risk of developing breast cancer.

### Knowledge of Cancer Risk Factors

Overall, the HINTS 6 participants included in our analysis had low knowledge about behavioral risk factors for cancer with the exception of alcohol consumption. A higher proportion of the Black respondents (50%) believed that drinking alcohol would have a significant impact on their chances of developing cancer compared to the White respondents (20%) (*p* = 0.028). In addition, both Black and White women seemed to be unaware of how dietary habits could impact their risk of getting cancer. For example, only 36.4% of the Black participants responded “A lot” to the question “How much do you think that not eating enough fruits and vegetables could increase a persons chance of developing cancer?” Our qualitative results confirmed the quantitative findings. Many of the qualitative phase participants understood that eating healthier and reducing their intake of junk food would prevent breast cancer, believing that “living a healthy lifestyle” and “making sure health and wellness is okay” would reduce their risk. However, most of the qualitative phase participants did not mention any specific foods or lifestyle behaviors that they thought would increase or decrease their chances of developing cancer and instead used “healthy” as a broad term.

### Need for Increased Breast Cancer Awareness Among Young Black Women

In addition, to asking about the key variables, qualitative phase participants were asked to share their recommendations for increasing awareness about and engagement in age-appropriate breast cancer screening. The participants suggested that there is a need to make breast cancer screening more accessible for young Black women. For example, one qualitative phase participant emphasized the importance of testing being accessible for everyone, saying we should “increase access to testing centers for breast cancer so people can catch it early on.” The participants also highlighted the importance of spreading awareness about breast cancer among young women to ensure that they have the information they need to decrease their risk of developing breast cancer. In addition, the qualitative phase participants noted that “teaching students about warning signs and how to self-check” was essential in helping increase young Black women’s chances of early detection of breast cancer.

## Discussion

We found that some cancer risk perceptions among young Black women were statistically significantly lower than those of young White women. Young Black women believed that it was difficult to know which recommendations to follow when trying to prevent cancer. Also, the majority of the Young Black women believed that there was not much that could be done to decrease the risk of developing cancer.

Young Black women have a significantly higher incidence of invasive breast cancer than White women [5, 24]. Lack of awareness about the primary and secondary prevention of breast cancer was common among young Black women in our study. This finding was similar to other studies that have found that Black women were less likely to perceive that they were at risk of getting cancer compared to White women [24]. Misconceptions about breast cancer risk factors were also apparent. Other studies have revealed that misconceptions about breast cancer screening act as a barrier to breast cancer screening among Black women [11, 25].

Cancer fatalism was common among our Black participants. Similarly, other studies examining cancer beliefs among Black women have found that cancer fatalism was associated with cancer information-seeking [26]. However, we found that the majority of our participants believed that the vast amount of different information about cancer screening guidelines makes it difficult for them to know which guidelines to follow. Currently, there are organizations that provide conflicting information about breast cancer screening in women under the age of 40. Therefore, there is a need to provide young Black women with information that will enable them to know which guidelines to follow according to their personal risk profiles. Rose et al. found that the provision of objective and understandable information about breast cancer was found to be a facilitator for breast cancer screening among Black women in a study by [27]. Having access to breast cancer screening guidelines that are understandable by young Black women may increase adherence to breast cancer screening guidelines for high-risk women.

The interview participants in our study provided common suggestions for ways to disseminate information about breast cancer, including through educational activities at colleges. Community-engaged strategies are needed to ensure that the messages and channels used to disseminate breast cancer education interventions targeted towards young Black women are effective.

### Implications

Our results are similar to other studies that have shown a lack of general cancer awareness and breast cancer–specific knowledge among young Black women [13, 16]. Young Black women have excessively high breast cancer mortality rates. Increased awareness of breast cancer risk factors and age-based screening guidelines may lead to reductions in late-stage breast cancer diagnoses and improved survival rates. For example, Pinheiro and her colleagues found that the odds of completing breast cancer screening were higher among women with high fatalistic cancer beliefs [28]. Population-specific breast cancer education interventions that are tailored to meet the health communication preferences of young Black women could act as a facilitator for age- and risk-appropriate breast cancer screening [29]. In addition, the implementation of multilevel interventions appears to have a positive impact on breast cancer mortality rates among Black women [30–34]. Therefore, further research is needed to determine how to develop multilevel approaches to increasing early detection of breast cancer in young Black women.

### Limitations

The use of a mixed methods study design was a major strength of this study. The study design enabled us to use multiple sources of data to investigate our research question. By conducting qualitative interviews, we were able to have in-depth conversations with young Black women that provided detailed responses to key questions about the breast cancer knowledge and risk perceptions. Our study is not without limitations. One significant limitation was the small number of young Black women who participated in the HINTS 6. Despite the use of a sampling strategy aimed at increasing the number of minority adult respondents, young Black women were severely underrepresented among the HINTS 6 participants [22]. Therefore, our sample size for the quantitative phase of the study was extremely small. In addition, the cancer belief section of the HINTS 6 did not include breast cancer–specific questions. As a result, we were limited to data about general cancer beliefs. The qualitative data was collected from Black women who were students at a large, public university in the southeast region of the US. Therefore, the results of the qualitative data analysis may not be transferable to Black women in other contexts.

## Conclusion

Young Black women have excessively high breast cancer mortality rates. The findings of our mixed methods study indicate that young Black women have a low perception of cancer risk, low knowledge about breast cancer risk factors, and have a need for simplified information about breast cancer screening guidelines. Our findings underscore the importance of developing, disseminating, and implementing breast cancer education interventions that are targeted towards young Black women.

## Data Availability

The HINTS 6 data is publicly available.
